# Dose-Dependent Cognitive Decline, Anxiety, and Locomotor Impairments Induced by Doxorubicin: Evidence from an Animal Model

**DOI:** 10.3390/biology13110939

**Published:** 2024-11-16

**Authors:** Ângela Amaro-Leal, Ana I. Afonso, Filipa Machado, Liana Shvachiy, Isabel Rocha, Tiago F. Outeiro, Vera Geraldes

**Affiliations:** 1 Egas Moniz Center for Interdisciplinary Research (CiiEM), Egas Moniz School of Health & Science, 2829-511 Almada, Portugal; 2 Faculdade de Medicina, Universidade de Lisboa, Av. Prof Egas Moniz, 1649-028 Lisbon, Portugal; 3 Centro Cardiovascular da Universidade de Lisboa, Faculdade de Medicina, Universidade de Lisboa, Av. Prof Egas Moniz, 1649-028 Lisbon, Portugal; 4 Department of Experimental Neurodegeneration, Center for Biostructural Imaging of Neurodegeneration, University Medical Center Göttingen, 37073 Göttingen, Germany; 5 Max Planck Institute for Multidisciplinary Sciences, 37075 Göttingen, Germany; 6 Translational and Clinical Research Institute, Faculty of Medical Sciences, Newcastle University, Newcastle Upon Tyne NE1 7RU, UK; 7 Scientific Employee with an Honorary Contract at Deutsches Zentrum für Neurodegenerative Erkrankungen (DZNE), 37075 Göttingen, Germany

**Keywords:** doxorubicin, anxiety, chemotherapy-induced cognitive impairment, cognitive dysfunction

## Abstract

Cognitive impairment and anxiety are common side effects of chemotherapy, particularly with the use of doxorubicin (DOX), known as “chemobrain”. This study aimed to examine the dose-dependent effects of DOX on cognitive decline, anxiety, and locomotor activity in healthy female Wistar rats. The rats were divided into groups receiving low (2 mg/kg), intermediate (4 mg/kg), and high (5 mg/kg) doses of DOX for four weeks, alongside a control group. Behavioral tests, including open field, elevated plus maze, and Y-maze tests, assessed anxiety, locomotion, and cognitive performance, while brain tissue analysis evaluated neuroinflammation using markers such as GFAP and Iba-1. The results showed that all doses of DOX induced anxiety-like behavior, reduced locomotion, and caused neuroinflammation in the hippocampus, with more severe effects at higher doses. Notably, high-dose DOX also caused short-term memory deficits. These findings highlight the dose-dependent nature of DOX’s impact on behavior and cognition, suggesting that DOX plays a key role in the development of cognitive symptoms during chemotherapy. Further research is needed to understand the mechanisms behind these effects and to explore potential interventions.

## Simple Summary

This study investigated the effects of doxorubicin, a chemotherapy drug, on behavior and cognition using a rat model. Doxorubicin is widely used to treat cancer, but it can also cause memory problems, anxiety, and reduced movement. The researchers aimed to understand how different doses of this drug affect these behaviors over time. They administered low, intermediate, and high doses of doxorubicin to healthy rats and monitored their anxiety, locomotion, and memory. The results showed that all doses led to anxiety, reduced movement, and memory loss, with the highest doses causing the most severe symptoms. The study also revealed that doxorubicin causes inflammation in the brain, which may explain these negative effects. These findings are important because they suggest that even standard doses of this chemotherapy drug can have serious side effects on mental health. Understanding these effects could help improve the quality of life for cancer patients undergoing treatment by finding ways to reduce or manage these symptoms. The study highlights the need for further research into protecting the brain from the side effects of chemotherapy.

## 1. Introduction

Cognitive impairment is one of the most detrimental consequences of chemotherapy in cancer patients, with a higher prevalence among women, ranging from 15% to 75%, and it often persists even after the conclusion of chemotherapy [1,2,3,4].

Doxorubicin (DOX), an anthracycline antineoplastic agent widely utilized in multi-drug chemotherapy regimens for solid tumor treatment, is well known for its potential to induce cognitive impairment. Its ability to penetrate the blood–brain barrier raises significant concerns regarding neurotoxicity, potentially leading to “chemobrain”, a condition linked to neuroinflammation and neuronal apoptosis [5]. Additionally, DOX has been shown to alter the gut microbiome, which may impact brain health through the gut–brain axis, thereby amplifying its systemic effects [6]. Patients with breast cancer undergoing DOX adjuvant therapy exhibit a decline in at least one neuropsychological test during and after standard-dose chemotherapy [7,8]. Although cognitive function may improve after treatment, those who experience a cognitive deficit (>50%) may endure lasting impairments in one or more cognitive domains [8].

Additionally, patients with breast cancer experience anxiety at various stages, such as during screening, waiting for results, receiving a diagnosis, undergoing treatment, or anticipating cancer recurrence [9]. Such situations increase anxiety levels, potentially leading to increased pain, sleep disturbances, nausea, vomiting, and diminished quality of life for these patients [9,10]. These deficits in attention, learning, memory, and anxiety, often termed “chemobrain”, significantly hinder day-to-day functioning and adversely impact the quality of life [2,4,11,12].

In rodent models, both acute and repeated DOX administration elicit behavioral disturbances and cognitive impairment, manifesting as decreased performance in diverse learning and memory tasks [13]. DOX treatment also triggers severe side effects in the nervous system, provoking neurobehavioral changes such as anxiety and depressive-like behavior [14]. Furthermore, cardiac alterations induced by DOX can disrupt various cognitive domains, affecting memory, executive function, and emotional factors such as anxiety and depression [15].

The precise mechanisms underlying chemotherapy-induced cognitive impairment, stemming from both the cancer itself and its treatment, remain poorly understood. These adverse effects are attributed to peripheral toxic effects, particularly in anthracycline regimens, which lead to downstream structural and functional brain alterations. These changes involve DNA damage, oxidative stress, inflammatory responses, dysregulation of apoptosis and autophagy, altered neurotransmitter levels, disturbances in essential kinases, mitochondrial dysfunction, interactions with glial cells, inhibition of neurogenesis, and epigenetic factors [5]. 

However, the cumulative dosage of DOX that triggers cognitive dysfunction, anxiety, and locomotor changes remains unclear. Therefore, in this experimental study using a healthy animal model, we aimed to elucidate the extent of the DOX’s various therapeutic doses impact on cognitive decline, anxiety, and locomotor activity. By delving into DOX’s effects on behavioral disturbances under cumulative treatment, we provide insights into the potential mechanisms underlying cognitive symptoms in humans undergoing DOX therapy.

## 2. Materials and Methods

All experimental procedures were in accordance with the European Community legislation on animal experimentation (Directive 2010/63/EU) and were approved by the Ethical Committee of the Academic Center of Lisbon (CAML) with reference number 394/17.

### 2.1. Model Development

Healthy female Wistar rats (n = 48), 12 weeks of age, were divided into four groups: low-dose DOX (LDOX, n = 12), intermediate-dose DOX (IDOX, n = 8), high-dose DOX (HDOX, n = 8), and CTL (n = 20). The DOX groups received weekly DOX injections (LDOX, 2 mg/kg; IDOX, 4 mg/kg; and HDOX, 5 mg/kg, i.p.) for 4 weeks. The CTL group was injected with saline solution and subjected to the same experimental protocol, except that behavioral tests were performed only once.

Animals were housed in the animal facility of the Faculty of Medicine of the University of Lisbon, with a maximum number of four animals per cage, with controlled temperature (22 ± 1 °C) and humidity (50 ± 5%) and synchronized under a 12/12 h light/dark cycle. Food (Mucedola, Settimo Milanese, Italy) and tap water (Epal, Lisbon, Portugal) were provided ad libitum. Although rats are nocturnal animals and are considerably more active at night, all testing occurred during the light phase of the light–dark cycle between 9 a.m. and 6 p.m.

### 2.2. Behavioral Evaluation

Behavioral tests were performed to assess changes in anxiety-like behavior, locomotor activity, and cognitive performance within an hour between tests to prevent interference [16]. All behavioral tests were conducted at 2 (T1) and 4 (T2) weeks after doxorubicin exposure. A digital video camera was used to track and record all the experiences using the i-SEC Guarding program (version 5.0.1.284). The performance of the animals was analyzed using the ANY-maze© software (Stoelting Co., Dublin, Ireland, version 4.82).

#### 2.2.1. Elevated Plus Maze

The elevated plus maze (EPM) test was used to evaluate anxiety-like behavior. The apparatus consists of a cross-like structure with four arms, two open and two closed (50 cm × 10 cm), raised 50 cm above the ground [17,18,19]. The animals were placed in the center and allowed to explore the maze for 5 min. The number of entries in the open/closed arms, time spent in the open/closed arms, distance (cm), and average speed (cm/s) were evaluated for each rat [20].

#### 2.2.2. Open Field Test

The open field test (OFT) is widely used to measure locomotor and exploratory activity and, more indirectly, anxiety [21,22]. The apparatus comprised a large square chamber 67 cm × 67 cm wide and 57 cm high. The animals were placed in the center of the square to explore the chamber for 5 min. Motor activity was evaluated by recording the total distance travelled and average velocity in the arena. The total number of entries in the three virtual areas was recorded to assess exploratory activity [23].

#### 2.2.3. Y-Maze Spontaneous Alternation

The Y-maze was used to access spatial memory [24]. It consists of an enclosed apparatus in three Y-shaped, identical arms with an angle of 120° between them (each arm is 35 cm long × 10 cm wide × 20 cm high). Rats were placed at the end of one arm and allowed to explore the maze for 8 min. The number of spontaneous alternations was recorded [24]. Additionally, the total number of arm entries for each animal was evaluated as a measure of locomotor activity [24].

### 2.3. Immunohistochemistry

At the end of the experimental protocol, the animals were sacrificed with an anesthesia overdose, and their brains were removed. The brains were kept at 4 °C overnight for post fixation in 4% paraformaldehyde (PFA) in phosphate buffer (pH 7.4). The brains were then immersed in increasing concentrations of sucrose (15% and 30%, Merck, Darmstadt, Germany in PBS 1× with sodium azide, Sigma, Darmstadt, Germany) and kept at 4 °C for later analysis. Freely floating coronal slices (25 μm) of the hippocampus (Bregma, –2.92 mm and –5.04 mm) were cut using a cryostat (Leica CM 3050S, Leica Microsystems, Wetzler, Germany). Immunohistochemistry was performed as previously reported [25,26,27]

Briefly, an antigen retrieval protocol [28] with permeabilization with 0.3% Triton X-100 (Sigma-Aldrich, Dorset, UK) and blocking with 5% normal goat serum (BioWest, Nuaillé, France) and 1% bovine serum (VWR, Radnor, PA, USA) was performed. Tissues were immunoassayed with NeuN (rabbit, abcam 1:200), GFAP (chicken, abcam, 1:500), and Iba-1 (goat, abcam, 1:200) (Abcam, Cambridge, UK) primary monoclonal antibodies and diluted in a blocking solution overnight at 4 °C. Following three TBS washes, sections were incubated with goat anti-rabbit IgG Alexa Fluor^®^ 568 (1:1000; Thermo Fisher, Waltham, MA, USA), goat anti-chicken IgG Alexa Fluor^®^ 633 (1:1000, Invitrogen, Waltham, MA, USA), and donkey anti-goat IgG Alexa Fluor^®^ 488 (1:1000; Thermo Fisher, Waltham, MA, USA)) secondary antibodies for 1 h at room temperature. Lastly, nuclei were counter-stained with DAPI (4′,6-diamidino-2-phenylindole, 1:10,000, Carl Roth, Karlsruhe, Germany). Sections were mounted in SuperFrost^®^ Microscope Slides using Fluoromount-G mounting media (Invitrogen, Darmstadt, Germany). Omission of the primary antibody resulted in no staining.

Z-stack images of the dentate gyrus region of the hippocampus were taken with a confocal point-scanning microscope (Zeiss LSM 900 with Airyscan 2, Carl Zeiss AG, Oberkochen, Germany) with a 20× objective (Objective Plan-Apochromat 20×/0.8) and tile scan to image the specific region of interest. Zen Microscopy Software (3.4 version, Carl Zeiss AG, Germany) was used for all imaging experiments, and the final images were post analyzed and quantified using Fiji open-source software (version 2.14.0/1.54f) [29,30,31,32].

### 2.4. Statistical Analyses

Box-plots of the values for each variable in the study represent the data. Statistical significance was set at *p* < 0.05. The normality distribution of the continuous variables was analyzed using the Kolmogorov–Smirnov test, and Levene’s test was used to assess the homogeneity of variance. Statistical differences were determined using a two-way ANOVA for both group and time factors (CTL vs. LDOX vs. IDOX vs. HDOX). Post hoc comparisons between groups and time points were performed to evaluate specific differences using Šídák’s multiple comparisons test. This approach allowed for the assessment of variations between the treatment groups and across the different time points while controlling for multiple comparisons. A one-way ANOVA with Dunnett’s multiple comparison test was used for molecular changes between CTL and DOX groups. All data were analyzed using GraphPad Prism 9 (GraphPad Software Inc., Boston, MA, USA).

## 3. Results

### 3.1. Behavioral Changes

#### 3.1.1. Higher DOX Dosages Induce Anxiety-like Behavior over Time

The effects of different DOX dosages on anxiety and stress levels in the EPM were tested at two different time points (T1: 2 weeks post DOX; T2: 4 weeks post DOX).

**Figure 1:**
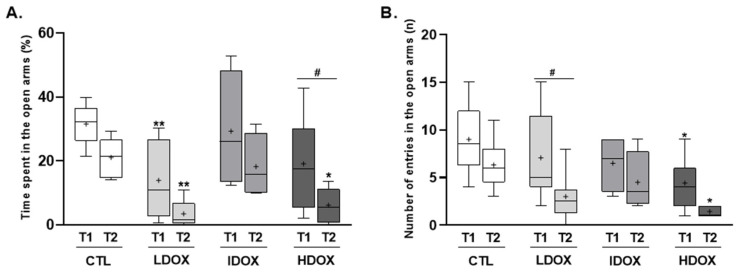
The effect of different DOX dosages on anxiety behavior was assessed using the EPM test. Box-plots of values of (**A**) the percentage of time and (**B**) the number of entries into the open arms. All variables were evaluated at two time points (T1 and T2). * *p* < 0.05 and ** *p* < 0.010 for comparison to CTL group and # *p* < 0.05 for intragroup comparison with two-way ANOVA with Šídák’s multiple comparisons test.

Two weeks post DOX, the animals exposed to different DOX doses spent significantly less time in the open arms (LDOX: 13.9 ± 3.5%, HDOX: 19.1 ± 4.9% vs. CTL: 31.6 ± 2.6%) and consequently more time in the closed arms (LDOX: 71.2 ± 5.3%, IDOX: 71.2 ± 6.8%, HDOX: 70.7 ± 7.5% vs. CTL: 42.6 ± 2.6%, of the EPM compared to the CTL group (Figure 1A, T1).

DOX treatments evoked a significant decrease in the percentage of time spent in the open arms (LDOX: 3.5 ± 1.2%, IDOX: 18.3 ± 5.0%, HDOX: 6.1 ± 1.9% vs. CTL: 21.1 ± 2.4%) 4 weeks post DOX and a significant increase in the time spent in the closed arms (LDOX: 66.6 ± 9.5% and HDOX: 85.3 ± 4.3% vs. CTL: 42.6 ± 2.6%) compared with CTL group (Figure 1A, T2).

In Figure 1A, the time spent in the open arms shows a dose-dependent decrease, particularly in the higher-dose group (HDOX), with a significant reduction at both T1 and T2 compared to controls. Although the intermediate dose (IDOX) appears to show a less pronounced effect than the low dose (LDOX) at T1, this could be attributed to individual variability or adaptive responses to DOX. However, by T2, the pattern aligns more closely with the expected dose response, with the HDOX group showing the most significant decrease (from T1 to T2: 19.1 ± 4.9% vs. 6.1 ± 1.9%, respectively). In panel B, the number of entries into the open arms follows a similar trend, with the HDOX group displaying the most significant reduction by T2 (from T1 to T2: 5.1 ± 0.9 vs. 1.1 ± 0.4, respectively), suggesting a cumulative impact of higher doses on anxiety-related behavior over time (Tables S1 and S2).

#### 3.1.2. DOX Induces Hypolocomotion and Hypoactivity

**Figure 2:**
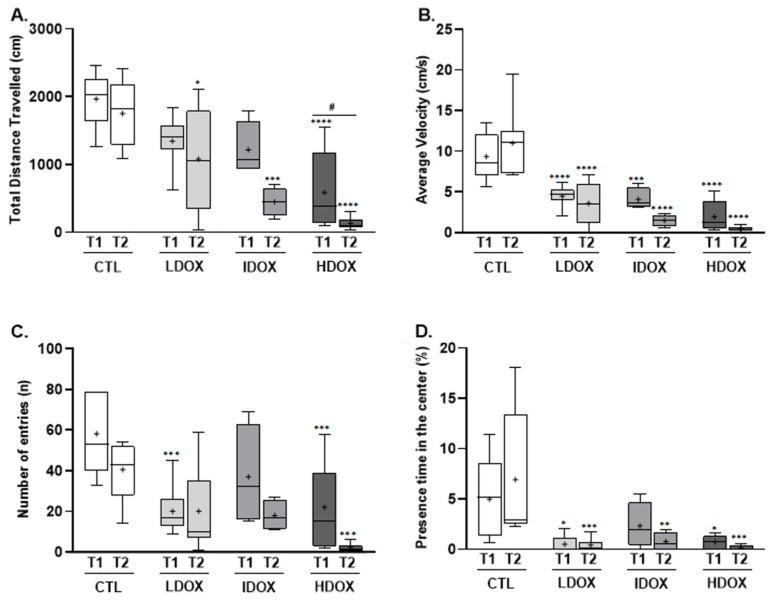
Effect of different DOX dosages on locomotion and exploratory activity assessed by OFT. Box-plots of values of (**A**) the total travel distance (cm), (**B**) the average velocity (cm/s), (**C**) the total number of entries in the three virtual zones, and (**D**) the percentage of time spent in the apparatus center. All variables were evaluated at two time points (T1 and T2). * *p* < 0.05, ** *p* < 0.01, *** *p* < 0.001, and **** *p* < 0.0001 for comparison to CTL group and # *p* < 0.05 for intragroup, with two-way ANOVA with Šídák’s multiple comparisons test.

The OFT was used to evaluate the locomotor and exploratory activities of the animals after DOX treatment (Figure 2). Animals exposed to the three different DOX dosages presented an extremely significant decrease (*p* < 0.001, *p* < 0.0001) in locomotor (total travelled distance and average velocity) and exploratory activity (total number of entries in the three virtual zones) compared to the CTL group at both time points evaluated (Figure 2A–C).

All animals (LDOX, IDOX, and HDOX groups) showed a significant decrease (*p* < 0.0001) in the % of time spent in the center of the apparatus when compared to the CTL group at both evaluated time points (Figure 2D), corroborating the results of the EPM test.

The HDOX dosage evoked a significant loss of locomotor (distance travelled: 586.8 ± 199.3 cm vs. 132.6 ± 30.2 cm; average velocity: 1.9 ± 0.7 cm/s vs. 0.4 ± 0.1 cm/s, Figure 2A,B) and exploratory activity (22.0 ± 7.8 vs. 2.0 ± 0.7, Figure 2C) as well as the % of presence time in the center of the apparatus (0.7 ± 0.2% vs. 0.1 ± 0.1%, Figure 2D) from T1 to T2. The IDOX dosage induced a significant decrease (distance travelled: 1015 ± 146.9 cm vs. 450 ± 104.4 cm and average velocity: 3.4 ± 0.5 cm/s vs. 1.5 ± 0.3 cm/s) in locomotor activity over time (Figure 2A,B; Tables S3–S6).

#### 3.1.3. Intermediate and Higher DOX Dosages Induce Short-Term Memory Impairments

**Figure 3:**
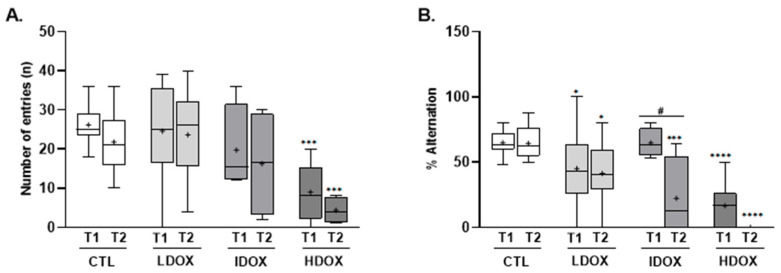
The effect of different DOX dosages on spatial learning and memory was assessed using the Y-Maze test. Box-plots of values of (**A**) the total number of entries in the three arms and (**B**) the number of spontaneous alternations. All variables were evaluated at two time points (T1 and T2). * *p* < 0.05, *** *p* < 0.001, and **** *p* < 0.0001 for comparison to CTL group and # *p* < 0.05 for intragroup, with two-way ANOVA with Šídák’s multiple comparisons test.

The effects of different DOX dosages on spatial working learning and memory were evaluated using the Y-maze spontaneous alternation test (Figure 3; Tables S7 and S8). The number of entries tended to decrease in the three DOX groups over time compared to animals not exposed to DOX, which was extremely significant (*p* < 0.001) for animals exposed to 20 mg of DOX (number of entries: 9.0 ± 2.5 vs. 4.4 ± 1.1; Figure 3A).

Figure 3B shows a significant decrease in the percentage of alternations over time in the high DOX dose (T1: 16.7 ± 6.2% vs. T2: 0.0 ± 0.0%) as well as the intermediate DOX dose group (T1: 64.7 ± 5.6% vs. T2: 22.3 ± 15.2%) compared to the CTL group (T1: 64.8 ± 2.9% vs. T2: 64.4 ± 3.8%).

### 3.2. Molecular Changes

#### DOX Increases the Number of Astrocytes and Microglia Without Neuroinflammation

**Figure 4:**
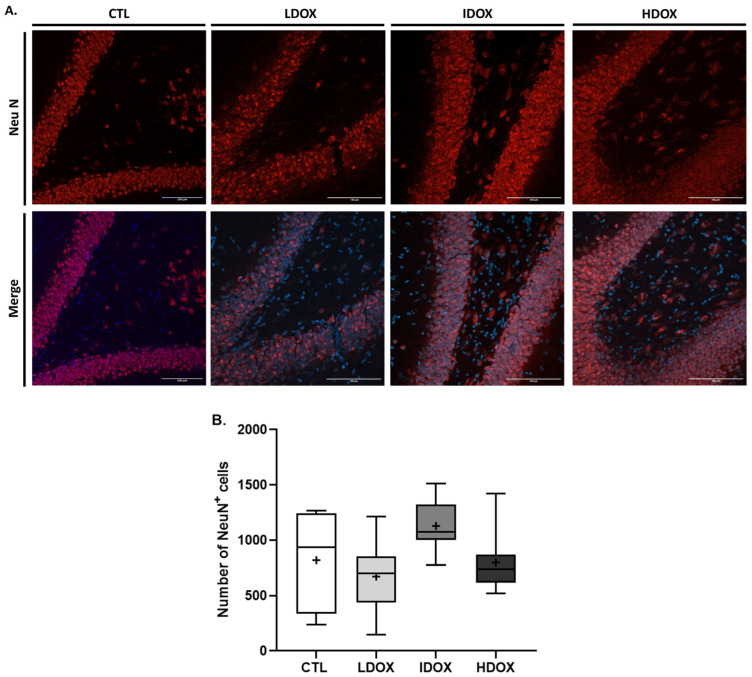
Effects of different DOX dosages on neuronal (NeuN) markers by immunohistochemistry. (**A**) Representative images of neurons stained with NeuN (1:500). (**B**) Box-plot of values of NeuN-positive cells. Images were acquired using a confocal point-scanning microscope (Zeiss LSM 900 with Airyscan) with a 20× objective. The scale bar is 100 µm for the stained images.

**Figure 5:**
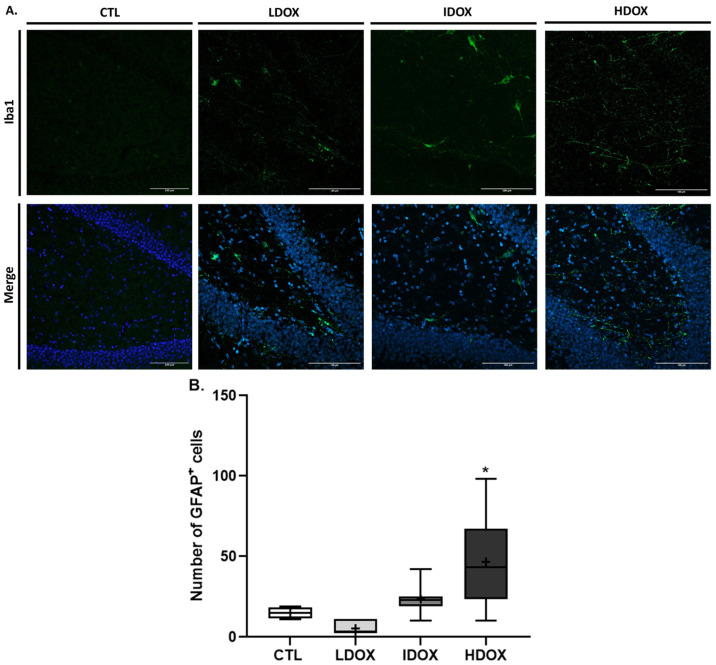
Effect of different DOX dosages on astrocytic (GFAP) markers by immunohistochemistry. (**A**) Representative images of glial fibrillary acidic protein (GFAP; 1:500)-stained astrocytes. (**B**) Box-plot of values of GFAP-positive cells. Images were acquired using a confocal point scanning microscope (Zeiss LSM 900 with Airyscan) with a 20× objective. The scale bar is 100 µm for stained images. The symbols denote statistically significant differences between groups (Ctrl vs. HDOX: * *p* < 0.05); one-way ANOVA with Dunnett’s multiple comparison test.

**Figure 6:**
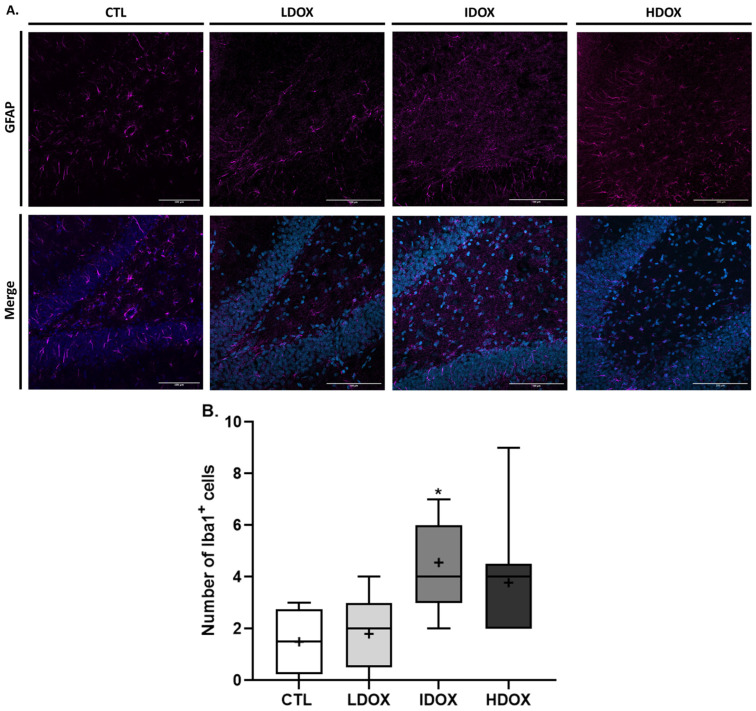
Effect of different DOX dosages on microglial (Iba1) markers by immunohistochemistry. (**A**) Representative images of Iba1 (1:250) microglia. (**B**) Box-plot of values of Iba1-positive cells. Images were acquired using a confocal point-scanning microscope (Zeiss LSM 880 with Airyscan) with a 20× objective. The scale bar is 100 µm for stained images. The symbols denote statistically significant differences between groups (Ctrl vs. IDOX: * *p* < 0.05); one-way ANOVA with Dunnett´s multiple comparison test.

To enhance the comprehension of behavioral alterations, a quantitative analysis was conducted on the brain tissue of female rats using immunohistochemistry. The dentate gyrus (DG) of the hippocampus was selected to study astrogliosis, microgliosis, and neurodegeneration since this area is associated with stress and depression [33,34]. Representative images and histograms for Neu-N (DG hippocampal area, positive cells in red, 20× objective, maximum intensity), GFAP (positive cells in green, 20× objective, z-stack), and Iba-1 (positive cells in green, 20× objective, z-stack) are shown in Figure 4, Figure 5, and Figure 6, respectively.

Quantitative analysis of NeuN-positive cells did not reveal significant changes in animals exposed to varying DOX dosages compared with the CTL group (Figure 4B, Table S9).

In addition to astroglia, intermediate DOX-treated animals showed a significant increase in the number of Iba1-positive cells compared to the control group (Figure 6B: CTL 1.5 ± 0.65 vs. IDOX 4.56 ± 0.58, *p* < 0.05). Although not statistically significant, the HDOX-treated group also displayed a rise in the number of Iba1-positive cells (Figure 6B: CTL 1.5 ± 0.65 vs. IDOX 3.78 ± 0.76, *p* > 0.05; Tables S10 and S11).

## 4. Discussion

Chemotherapy, which offers significant therapeutic benefits, has been increasingly associated with the development of neuropsychological disorders in patients, manifesting as cognitive symptoms such as confusion, memory loss, and difficulties with attention and concentration [35,36]. Of note, these cognitive impairments, commonly referred to as “chemobrain”, tend to affect women more frequently and can persist even after accounting for factors such as anxiety, depression, fatigue, and perceived cognitive dysfunctions [1,4,8,37]. This suggests that the neurotoxic effects of chemotherapeutic agents like doxorubicin (DOX) extend beyond immediate side effects and may contribute to long-term cognitive decline.

To elucidate the underlying mechanisms contributing to these cognitive and behavioral issues, animal models serve as invaluable resources for studying human diseases and uncovering disease prevention and treatment avenues [38]. In this study, we conducted a novel comparison of different doses of DOX in healthy female Wistar rats over four weeks, focusing on anxiety-like behavior, locomotor activity, and spatial memory. Our findings demonstrate that DOX-induced behavioral alterations are both dose- and time-dependent, underscoring the need to understand these dynamics within the framework of chemotherapy-related cognitive dysfunction. This study provides a clearer view of DOX’s dose-dependent neurotoxic effects, particularly regarding cognitive impairments linked to “chemobrain”. By pinpointing specific neuroinflammatory pathways in the hippocampus, our results shed light on the underlying mechanisms of DOX-induced cognitive and behavioral deficits. Additionally, this research suggests that interventions such as anti-inflammatory agents may mitigate these effects, offering valuable insights for enhancing patient care in cancer therapy.

All DOX dosages resulted in decreased locomotion accompanied by anxiety-like behavior, with intermediate and high doses leading to the most severe effects on the assessed behavioral parameters. Furthermore, high-dose DOX treatment was associated with a progressive decline in short-term memory, underscoring the exacerbation of cognitive impairment. This aligns with previous research demonstrating the neurotoxic effects of DOX, particularly at higher doses, which can lead to impairments across various cognitive domains, including memory. The observed decline in short-term memory is consistent with the established neurotoxic profile of DOX, emphasizing the need for careful consideration of dosage in therapeutic contexts.

The behavioral impact of DOX dosage was evident across various indicators, including decreased time in and fewer entries into the open arms of the elevated plus maze (EPM) and reduced time spent in the central square in the open field test. These behavioral outcomes underscore the intricate relationship between anxiety and locomotor activity, suggesting that anxiety expression is tightly linked to motor function impairment [39,40]. In this context, anxiety expression cannot be separated from the decline in locomotor activity, which is consistent with the pattern observed in the open field test when doxorubicin is present [39,41].

Additionally, we observed other behavioral changes that further indicate heightened anxiety levels. These included freezing, reduced movement, and thigmotaxis, where the animals preferred the edges of the testing arenas. These observations reflect significant alterations in their emotional processing [42]. Excessive anxiety, while a natural response that aids in adaptive survival, can interfere with normal brain function and diminish the behavioral flexibility necessary for effective adaptation [43]. Our current findings suggest that the neurotoxic effects of DOX may extend to disrupt neural circuits responsible for anxiety regulation, with particular involvement of the medial prefrontal cortex and the amygdala. [43]. The amygdala’s critical role in processing fear and anxiety, combined with the regulatory functions of the prefrontal cortex, indicates that DOX treatment may lead to significant disturbances in the neural circuitry governing anxiety and emotional regulation [44]. This disruption can compromise the behavioral flexibility required for adaptive responses to stressors, further complicating the cognitive and emotional landscape of patients undergoing chemotherapy [44].

Moreover, our findings support the notion that the alterations in anxiety behavior induced by DOX exposure correlate with changes in hippocampal gene expression, as reported in previous studies [45].

Rats treated with DOX consistently exhibited signs of anxiety in various testing paradigms, shown by an increased percentage of time spent and a higher number of entries into the maze arms compared to the control group. This observation also confirmed that DOX treatment decreased the total distance traveled and reduced average speed in the OFT and EPM, indicating suppressed locomotor activity across all tested DOX doses—a characteristic sign of disease behavior [46].

Our results align with the findings of Aziriova et al., who demonstrated that cumulative administration of doxorubicin at a dose of 20 mg/kg increased anxiety levels in behavioral tests, including the OFT, EPM, and the light–dark box. Additionally, they noted a significant increase in systolic blood pressure and oxidative stress parameters in rats exposed to DOX for four weeks [14]. Similarly, Merzoug et al. reported anxiety-like behavior characterized by impaired locomotion and exploratory activities in rats 72 h after receiving a cumulative doxorubicin dose of 15 mg/kg [47]. These findings suggest that acute administration of a clinical DOX dose yields comparable effects to our intermediate and chronic DOX administration, highlighting the potential for behavioral dysfunction to persist and worsen with continued treatment [47].

In addition to its impact on locomotor and anxiety-related behaviors, DOX-induced cognitive impairment is a significant concern, given its adverse effects on the quality of life of cancer survivors [48,49]. The results of the Y-maze spontaneous alternation test indicated that animals exposed to a high doxorubicin dosage (HDOX) exhibited a notable decline in spatial learning performance, as evidenced by a reduction in the percentage of spontaneous alternations, along with a decrease in the number of arm choices and the number of correct responses. Conversely, animals subjected to LDOX and IDOX dosages displayed a tendency toward diminished spatial learning performance. These deficits align with the critical role of the hippocampus in spatial processing and memory formation, further highlighting how DOX administration can compromise cognitive function, including episodic and spatial memory [37,50]. Previous studies emphasize the potential of DOX to induce hippocampus-dependent deficits in learning and memory, suggesting a mechanistic link between chemotherapy and cognitive decline [4,15,37,51].

An important limitation of this study concerns the one-hour intervals used between behavioral assays, which were scheduled to accommodate treatment-related logistical requirements. Although optimal practice typically involves longer inter-test intervals, to minimize stress-related carryover effects and ensure distinct behavioral assessments, the present study employed a shorter timeline to synchronize with treatment administration. In order to reduce this interference, tests were conducted sequentially in an order designed to minimize cumulative stress impacts. Thus, the distinct behavioral domains were assessed in a progressive sequence to reduce interference across measures.

This sequential approach was carefully chosen to balance the need for consistency and reliability across tests with the constraints of a condensed timeline, drawing on previously validated protocols [16]. Therefore, while our approach enabled comprehensive behavioral profiling within the study’s constraints, future work with extended inter-test intervals may provide more robust insights into the independent effects of each assay.

Another noteworthy aspect was the significant body weight loss observed across all three DOX dosage groups, which is indicative of systemic effects beyond cognitive and behavioral changes (data not presented here). Notably, animals exposed to intermediate and high doses of DOX exhibited comparatively less weight loss, frequently accompanied by the development of abdominal ascites. These findings are consistent with previous studies reporting both a decrease in body weight and the occurrence of ascites as associated effects of DOX treatment [52,53], underscoring the importance of considering the systemic effects of DOX beyond cognitive and behavioral changes when interpreting the study results.

Weight loss and ascites may independently influence locomotion and anxiety-like behaviors in the open field (OF) and elevated plus maze (EPM) tests. Nevertheless, evidence suggests that DOX-induced neuroinflammation and hippocampal alterations may also contribute to these behavioral changes [37]. Thus, while systemic effects are likely contributors, hippocampal involvement remains a plausible factor, warranting further investigation to better delineate these influences.

The pathological behavioral alterations induced by DOX are associated with oxidative stress and myelosuppression [47,54]. Oxidative stress leads to the production of harmful reactive oxygen species, which can damage cellular components and disrupt normal brain function, potentially contributing to behavioral alterations. Additionally, DOX causes myelosuppression, leading to a reduction in the production of blood cells in the bone marrow. This can result in systemic effects including fatigue and weakness, which may contribute to the observed behavioral changes. Moreover, oxidative/nitrosative stress may play a causal role in these behavioral changes by affecting biochemical processes in normal, non-cancerous cells [55]. Both oxidative and nitrosative stresses have been shown to affect biochemical processes in normal cells, including those in the brain. These processes include alterations in signaling pathways, changes in gene expression, and disruption of cellular metabolism. Such alterations can ultimately lead to neuronal damage, neuroinflammation, and synaptic dysfunction, processes that are associated with anxiety, depression, and cognitive impairment.

Neuroinflammation promotes neurodegenerative processes and contributes to cognitive impairment and behavioral disturbances, suggesting a potential mechanism by which DOX may induce these adverse effects in patients undergoing chemotherapy [54,55,56,57].

The activation of astrocytes and microglial cells in the hippocampus of rats treated with DOX, as evidenced by increased expression of GFAP and Iba1, indicates the presence of astrogliosis and microgliosis, which are hallmark features of neuroinflammation. The current findings align with the research conducted by Moretti et al., revealing elevated expression of GFAP and Iba1 in the frontal cortex, hypothalamus, and hippocampus after doxorubicin exposure [45]. Neuroinflammation promotes neurodegenerative processes and contributes to cognitive impairment and behavioral disturbances, suggesting a potential mechanism by which DOX may induce cognitive impairment and behavioral disturbances in patients undergoing DOX therapy [56,57,58,59].

The lack of statistical significance in the HDOX group may be attributed to the limited sample size, which could mask more subtle effects on microglial cell activation. Future studies with larger sample sizes could provide a more comprehensive understanding of these trends.

## 5. Conclusions

This study highlights the complex interplay between DOX treatment and its behavioral, cognitive, and physiological consequences. By examining these relationships, we demonstrated the dose-dependent impact of doxorubicin (DOX) on cognitive and behavioral functions, with the most severe effects observed in the Wistar rats receiving 20 mg/kg of the drug. The findings highlight significant impairments in cognition and anxiety responses, particularly after exposure to doses as low as 8 mg/kg. Impaired locomotor and exploratory activities were noted in all DOX-treated animals, indicating that even lower doses can disrupt movement and behavior. Furthermore, DOX-induced neuroinflammatory changes in the hippocampus, marked by increased astrocyte and microglial activity, suggest that neuroinflammation plays a key role in the cognitive and behavioral disturbances observed.

Future research should explore the molecular mechanisms of DOX-induced neuroinflammation and cognitive impairment, particularly on long-term effects. Investigating neuroprotective strategies such as anti-inflammatory agents or antioxidants may help prevent or mitigate these adverse outcomes. This study supports the hypothesis that DOX significantly contributes to cognitive symptoms in chemotherapy patients, emphasizing the need for further research into the interplay between DOX exposure, cancer treatment, and neuroinflammation to develop effective interventions for “chemobrain”.

## Data Availability

Data are contained within the article and Supplementary Materials.

## Supplementary Materials

The following supporting information can be downloaded at: https://www.mdpi.com/article/10.3390/biology13110939/s1, Table S1: The effect of different DOX dosages on anxiety behavior was assessed using the EPM test on the percentage of time spent in the open arms; Table S2: The effect of different DOX dosages on anxiety behavior was assessed using the EPM test on the number of entries into the open arms; Table S3: Effect of different DOX dosages on locomotion and exploratory activity assessed by OFT on the Total travelled Distance (cm); Table S4: Effect of different DOX dosages on locomotion and exploratory activity assessed by OFT on the average velocity; Table S5: Effect of different DOX dosages on locomotion and exploratory activity assessed by OFT on the total number of entries in three virtual zones; Table S6: Effect of different DOX dosages on locomotion and exploratory activity assessed by OFT on the percentage of time spent in the center; Table S7: The effect of different DOX dosages on spatial learning and memory was assessed using the Y-Maze test on the total number of entries in the three arms; Table S8: The effect of different DOX dosages on spatial learning and memory was assessed using the Y-Maze test on the number of spontaneous alternations; Table S9: Effects of different DOX dosages on neuronal (NeuN) markers by immunohistochemistry; Table S10: Effects of different DOX dosages on astrocytic (GFAP) markers by immunohistochemistry; Table S11: Effects of different DOX dosages on microglial (Iba1) markers by immunohistochemistry.
