# Supplementary material for: Dose-Dependent Cognitive Decline, Anxiety, and Locomotor Impairments Induced by Doxorubicin: Evidence from an Animal Model
Source: Biology (Basel). 2024 Nov 16;13(11):939. doi: 10.3390/biology13110939 (PMC11592173; doi:10.3390/biology13110939)
Supplement: Supplementary file 1 [file biology-13-00939-s001.zip › biology-3251508-supplementary.pdf]

Supplementary Table S1. The effect of different DOX dosages on anxiety behavior was assessed using the EPM test on the percentage of time spent in the open arms in the CTL, LDOX, IDOX, and HDOX groups evaluated at two-time points (T1 and T2). The table includes details of the statistical analysis conducted, presenting ANOVA results for interactions between Time and Group, main effects of Time and Group, as well as corresponding F values, degrees of freedom (DF), mean squares (MS), and associated p-values.

% of time spent in the open arms

ANOVA table	SS	DF	MS	F (DFn, DFd)	P value
Time x Group	16,83	3	5,610	F (3, 23) = 0,08246	P=0,9689
Time	1543	1	1543	F (1, 23) = 22,68	P<0,0001
Group	2905	3	968,3	F (3, 23) = 7,708	P=0,0010

Supplementary Table S2. The effect of different DOX dosages on anxiety behavior was assessed using the EPM test on the number of entries into the open arms in the CTL, LDOX, IDOX, and HDOX groups evaluated at two different time points (T1 and T2). The table includes details of the statistical analysis conducted, presenting ANOVA results for interactions between Time and Group, main effects of Time and Group, as well as corresponding F values, degrees of freedom (DF), mean squares (MS), and associated p-values.

The Number of Entries into the Open Arms

ANOVA table	**SS**	**DF**	**MS**	**F (DFn, DFd)**	**P value**
Time x Group	8,530	3	2,843	F (3, 25) = 0,2467	P=0,8629
Time	107,4	1	107,4	F (1, 25) = 9,317	P=0,0053
Group	146,3	3	48,78	F (3, 25) = 6,502	P=0,0021

Supplementary Table S3. Effect of different DOX dosages on locomotion and exploratory activity assessed by OFT on the Total travelled Distance (cm) in the CTL, LDOX, IDOX, and HDOX groups evaluated at two-time points (T1 and T2). The table includes details of the statistical analysis conducted, presenting ANOVA results for interactions between Time and Group, main effects of Time and Group, corresponding F values, degrees of freedom (DF), mean squares (MS), and associated p-values.

Total travelled Distance (cm)

ANOVA table	**SS**	**DF**	**MS**	**F (DFn, DFd)**	**P value**
Time x Group	467365	3	155788	F (3, 25) = 0,9434	P=0,4346
Time	2207274	1	2207274	F (1, 25) = 13,37	P=0,0012
Group	15202759	3	5067586	F (3, 25) = 15,19	P<0,0001

Supplementary Table S4. Effect of different DOX dosages on locomotion and exploratory activity assessed by OFT on the average velocity in the CTL, LDOX, IDOX, and HDOX groups evaluated at two different time points (T1 and T2). Table detail the values of number, minimum, 25% percentile, median, 75% percentile, maximum, range, mean, standard deviation, standard error of the mean, lower 95% confidence interval of the mean, and upper 95% confidence interval of the mean.

The Average Velocity

ANOVA table	**SS**	**DF**	**MS**	**F (DFn, DFd)**	**P value**
Time x Group	37,20	3	12,40	F (3, 30) = 3,022	P=0,0450
Time	9,737	1	9,737	F (1, 30) = 2,373	P=0,1339
Group	831,5	3	277,2	F (3, 30) = 42,30	P<0,0001

Supplementary Table S5. Effect of different DOX dosages on locomotion and exploratory activity assessed by OFT on the total number of entries in three virtual zones in the CTL, LDOX, IDOX, and HDOX groups evaluated at two different time points (T1 and T2). The table includes details of the statistical analysis conducted, presenting ANOVA results for interactions between Time and Group, main effects of Time and Group, as well as corresponding F values, degrees of freedom (DF), mean squares (MS), and associated p-values.

Total number of entries in three virtual zones

ANOVA table	**SS**	**DF**	**MS**	**F (DFn, DFd)**	P value
Time x Group	1179	3	393,0	F (3, 23) = 1,702	P=0,1944
Time	2312	1	2312	F (1, 23) = 10,01	P=0,0043
Group	8846	3	2949	F (3, 23) = 10,28	P=0,0002

Supplementary Table S6. Effect of different DOX dosages on locomotion and exploratory activity assessed by OFT on the percentage of time spent in the center in the CTL, LDOX, IDOX, and HDOX groups evaluated at two different time points (T1 and T2). The table includes details of the statistical analysis conducted, presenting ANOVA results for interactions between Time and Group, main effects of Time and Group, corresponding F values, degrees of freedom (DF), mean squares (MS), and associated p-values.

% of time spent in the center

ANOVA table	SS	DF	MS	F (DFn, DFd)	P value
Time x Group	15,54	3	5,179	F (3, 21) = 0,7452	P=0,5372
Time	0,06041	1	0,06041	F (1, 21) = 0,008693	P=0,9266
Group	231,7	3	77,22	F (3, 21) = 11,93	P<0,0001

Supplementary Table S7. The effect of different DOX dosages on spatial learning and memory was assessed using the Y-Maze test on the total number of entries in the three arms in the CTL, LDOX, IDOX, and HDOX groups evaluated at two different time points (T1 and T2). The table includes details of the statistical analysis conducted, presenting ANOVA results for interactions between Time and Group, main effects of Time and Group, corresponding F values, degrees of freedom (DF), mean squares (MS), and associated p-values.

Total number of entries in the three-arms

**ANOVA table**	SS	DF	MS	F (DFn, DFd)	P value
**Time x Groups**	46,67	3	15,56	F (3, 30) = 0,6197	P=0,6077
**Time**	161,8	1	161,8	F (1, 30) = 6,445	P=0,0165
Groups	3559	3	1186	F (3, 30) = 8,452	P=0,0003

Supplementary Table S8. The effect of different DOX dosages on spatial learning and memory was assessed using the Y-Maze test on the number of spontaneous alternations in the CTL, LDOX, IDOX, and HDOX groups evaluated at two different time points (T1 and T2). The table includes details of the statistical analysis conducted, presenting ANOVA results for interactions between Time and Group, main effects of Time and Group, corresponding F values, degrees of freedom (DF), mean squares (MS), and associated p-values.

The number of spontaneous alternations

**ANOVA table**	**SS**	**DF**	**MS**	**F (DFn, DFd)**	**P value**
**Time x Groups**	2965	3	988,5	F (3, 30) = 2,159	P=0,1136
**Time**	3593	1	3593	F (1, 30) = 7,848	P=0,0088
**Groups**	28360	3	9453	F (3, 30) = 41,68	P<0,0001

Supplementary Table S9. Effects of different DOX dosages on neuronal (NeuN) markers by immunohistochemistry. NeuN-positive cells quantification in the CTL, LDOX, IDOX, and HDOX groups. The table includes details of the statistical analysis conducted, presenting ANOVA results for interactions between Groups, corresponding F values, degrees of freedom (DF), mean squares (MS), and associated p-value.

NeuN-positive cells quantification

ANOVA table	**SS**	**DF**	**MS**	**F (DFn, DFd)**	**P value**
Treatment (between columns)	888264	3	296088	F (3, 25) = 3,067	P=0,0463

Supplementary Table S10. Effects of different DOX dosages on astrocytic (GFAP) markers by immunohistochemistry. GFAP-positive cells quantification in the CTL, LDOX, IDOX, and HDOX groups. The table includes details of the statistical analysis conducted, presenting ANOVA results for interactions between Groups, corresponding F values, degrees of freedom (DF), mean squares (MS), and associated p-value.

GFAP-positive cells quantification

ANOVA table	**SS**	**DF**	**MS**	**F (DFn, DFd)**	**P value**
Treatment (between columns)	5489	3	1830	F (3, 19) = 6,428	P=0,0034

Supplementary Table S11. Effects of different DOX dosages on microglial (Iba1) markers by immunohistochemistry. Iba1-positive cells quantification in the CTL, LDOX, IDOX, and HDOX groups. The table includes details of the statistical analysis conducted, presenting ANOVA results for interactions between Groups, corresponding F values, degrees of freedom (DF), mean squares (MS), and associated p-value.

Iba1-positive cells quantification

ANOVA table	**SS**	**DF**	**MS**	**F (DFn, DFd)**	**P value**
Treatment (between columns)	40,42	3	13,47	F (3, 23) = 3,894	P=0,0219
